# Mindfulness‐based practices with family carers of adults with learning disability and behaviour that challenges in the UK: Participatory health research

**DOI:** 10.1111/hex.12914

**Published:** 2019-06-11

**Authors:** Tina Cook, Steven Noone, Megan Thomson

**Affiliations:** ^1^ Department of Disability and Education Liverpool Hope University Liverpool UK; ^2^ Northumberland, Tyne and Wear NHS Foundation Trust St. Nicholas Hospital Newcastle upon Tyne UK

**Keywords:** acceptance and commitment therapy, behaviour that challenges, facilitation, family carers, learning disability, mindfulness, participatory health research

## Abstract

**Background:**

Family carers of adults with learning disability and behaviours that challenge lead complex and stressful lives. Their caring role can leave them isolated and unsupported. In the UK, effective services designed to build resilience for people in long‐term caring roles are lacking. There are none (to our knowledge) designed using a participatory health research (PHR) approach with family carers and professionals.

**Objective:**

With positive behaviour support (PBS) and mindfulness and acceptance and commitment therapy (ACT) as key elements, a PHR approach was used to understand the basis for a successful course that supported the capabilities and resilience of family members in long‐term caring roles.

**Design:**

The research was guided by the principles of PHR with participation as the defining principle throughout. Central to the research were reflexive conversations (communicative spaces) where diverse knowledges were shared and critiqued.

**Findings:**

Mindfulness/ACT can change long‐standing response behaviours and build personal resilience and improve mental health. Elements enabling positive change included a facilitation approach for collaborative reflexivity and the complementary, interactive approach to collaborative enquiry for learning and decision making afforded by PHR.

**Discussion:**

The use of PHR accessed knowledges that would have been lost to more traditional, professional‐expert driven processes and facilitated change in constructs for action for both professionals and family carers. Findings challenge service providers to consider how experiential knowledge has agency in professional practice and service design. Reflection on the PHR process across the FaBPos project led to a re‐consideration of quality issues in relation to PHR and participation.

## 1 INTRODUCTION

Approximately 170 000 households in England live with an adult family member with a learning disability and behaviour that challenges.^1^ Studies carried out in the UK and Germany have demonstrated the strong commitment of family carers to providing excellent emotional, social and physical care for family members.^2^, ^3^, ^4^ These studies also highlight the on‐going, long‐term stress families experience due to constant juggling of day‐to‐day home life, coping with their family member's unpredictable behaviours and battles with inadequate services. A systematic review and synthesis of 15 research studies^2^ revealed a gap in support for long‐term family carers of people with learning disability and challenging behaviours.

Originating from mindfulness‐based stress reduction courses developed in the late 1970s,^5^ mindfulness‐based programmes (MBPs) have been adapted for a variety of clinical populations.^6^ In 2006, a 12‐week MBP for mothers of *children* with autism^7^ found an increased sense of satisfaction in parenting skills and a decrease in challenging behaviour amongst their children. Higher levels of mindfulness, acceptance and self‐compassion appear to reduce the impact of challenging behaviours on parental stress, anxiety and depression.^8^, ^9^ In 2019, a 30‐week MBP with mothers of *adolescents* with ASD, or with intellectual disability, also reported significant reductions in their levels of stress and reductions in disruptive behaviours amongst their sons/daughters.^10^ There are no known studies that examine the impact of MBPs with family carers of *adults* with a learning disability and no known intervention protocol for training‐based support activities for building their resilience.^11^

In the UK, the term learning disability describes an impairment of general mental abilities (typically measured as an IQ of 70 or below) along with a significant impairment of social and adaptive functioning. The terms learning disability and learning difficulties are often used interchangeably. In this paper, we have used the term learning disability as it was the term commonly used by most people involved in the project. Some self‐advocacy organizations argue, however, for the use of learning difficulty “to get across the idea that our learning support needs change over time”.^12^ We acknowledge and respect that perspective on learning potential.

The combination of mindfulness and acceptance and commitment therapy (Mindfulness/ACT) differs from traditional cognitive behavioural therapy. Rather than trying to teach people how to control their thoughts and feelings, it teaches people to notice, accept and embrace these, including previously unwanted ones. The current cognitive model for explaining how mindfulness and ACT work together suggests they may alter the relationship with stressful thoughts that evoke powerful emotional responses.^13^ The aim of the Family Based Positive Support (FaBPos) project was to understand the core components for an effective Mindfulness/ACT course for family carers of adults with learning disabilities and behaviour that challenges. Through the processes of participatory health research (PHR), the intention was to bring together the different sets of knowledge held by clinical psychologists (the facilitators) and family carers firstly to investigate the underpinnings for an effective course to support and develop resilience in the face of long‐term stress and secondly to investigate the impact of collaborative research engagement (PHR) as a process.

## 2 PARTICIPATORY HEALTH RESEARCH

In the UK, health research funders generally expect researchers to include the voices of patients in their projects.^14^ A national advisory group for patient and public involvement (PPI) in research, INVOVLE, was established in 1996 to support active PPI in NHS, public health and social care research.^15^ The term PPI is, however, applied to a broad range of engagement processes, from research that might engage the public as members of a research steering committee to the co‐creation of research led by, and for, those whose lives are directly affected by such studies. The fundamental difference between the two examples given above is that the former is driven and led from an external, professional, more distanced standpoint and the latter, PHR, has, as a key aim, the maximization of the participation of those whose life or work is the subject of the research in all stages of the research process.^16^

In recent years, there has been increased adoption of research approaches that move beyond collecting expert testament to embedding the knowledge of those whose lives/work are the subject of the study as a fundamental element of the research process.^17^ This goes beyond service user involved, or service user led research in that it values and uses multiple ways of seeing as the core driver for the research. No single form of knowledge, such as academic knowledge or practitioner knowledge, is given primacy. New knowledge is produced collectively rather than unilaterally by a particular subset of individuals. Agency is provided to the voices of those with lived experience in understanding and shaping the research, alongside more traditional knowledge bearers (professionals and practitioners). Embedding “popular knowledge”^18^ challenges the historical hierarchical view where professional knowledge is valued above situational and experiential knowledge, where professional observations are viewed as objective but service‐user perceptions viewed as subjective.^19^ If the subjective is considered less worthy or less important, this creates barriers to shared learning. It contributes to the gap between what is needed by service users and what is provided by services and historic notions of the professional as the knower and the service user as receiver of knowledge continue to shape how services are configured and delivered.^21^, ^22^, ^23^

The intention of the FaBPos project was for people to work together to build new knowledge for action, through research processes that involved family carers and professionals in shared looking, acting, critical reflection and decision making.

## 3 PREPARING FOR THE FaBPos PROJECT

The original project design team (PDT) consisted of a consultant clinical psychologist with a wealth of experience using Mindfulness/ACT, a university academic with experience as a practitioner working with family carers and of facilitating PHR, three family carers and a research assistant. Prior to undertaking the research, the team consulted with family carers in the local region about their caring experiences. They discussed what might support them in their caring role, what that support might look like and how they looked after themselves. Discussions with 4 mothers, 1 sister, 1 grandmother and 2 couples (mother and father) were held in their own homes. 4 group discussions were held in family carer centres with 12‐25 family carers per group. Informal conversations were also held between family carer members of the PDT and family carers they associated with. Learning from these consultations, outlined in Table 1, was then put together with the work of the PDT team and contributed to the design of the research project.

**Table 1:** Learning drawn from pre‐course consultations

Family carer perceptions of their role	Maintaining a good quality of life for the person they cared for was priority, often at the expense of their own quality of life. Both family carers themselves, and services, paid little attention to the well‐being of family carers
Family carer experiences	24/7 care is very hard and takes a toll on physical and mental health. This gets more pronounces with age. Most family carers had taken part in courses delivered by professionals and/or third‐sector organizations. They had not alleviated stress and did not foster space to build resilience. They had little confidence that future courses would help them. Being subjected to tests in relation to their lifestyles and mental health during previous engagements was described as being intrusive, an additional burden, and made them feel negative about themselves. They would not take part in a course that used such approaches. Time was very precious with time constraints a major factor in adding to their stress. Given their caring responsibilities, family carers considered full‐day sessions would be too long
Family carer views and expectations of a course	They wondered whether family carers would recognize/prioritize the need to come to something that had a strong focus on themselves rather than their relatives. There was suspicion of the idea of Mindfulness/ACT and whether families could be convinced that it was worth trying. Some wanted to access informal support where they could talk with others rather than attend a course. Some wanted their knowledge to be recognized, valued and used in any course. They would like to help other families if they could. This would be a rationale for attending a course, to be with others in a practical way

The project gained ethical approval from both University and NHS ethical governance processes. In the time, it took to gain funding and acquire the related ethical approvals; however, changes in the lives of the family carers and the research assistant meant they were no longer able to be researchers on the project. Two family carers remained connected as members of the Steering Group/Advisory Committee, and a new research assistant was recruited.

## 4 RESEARCH DESIGN

**Figure 1:**
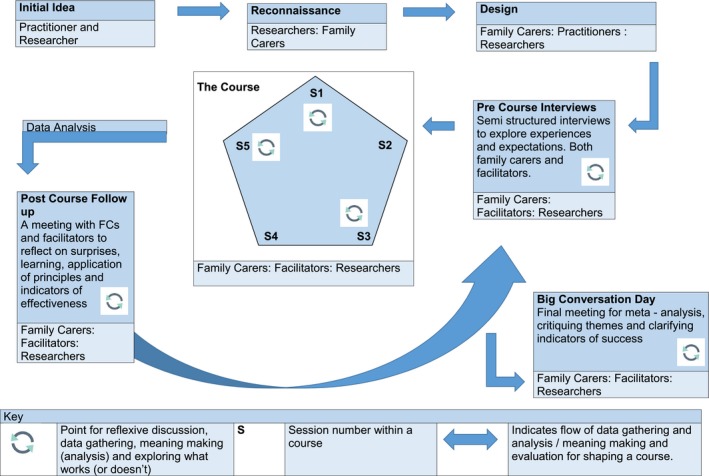
Action research cycle

The underpinning design for the research was a series of three courses of Mindfulness/ACT. Each course consisted of five sessions of Mindfulness/ACT. These were hosted in non‐NHS venues with iterative phases of relationally based dialogical engagements (communicative spaces) at the core of the research process. This created the basis for an action research cycle (Figure 1) where everyone involved, shaped both the design and content of the course, generated data and made meaning (data analysis) from that data.

**Table 2:** Description of participants

	Number	Description
Family carers	18	1 male and 17 females aged between 30 and 70. They came from all walks of life. Many had other caring responsibilities, for instance caring for elderly parents. Only 3 were known to be in employment at the start of their course: 1 full time and 2 part time (1 was about to be made redundant, partly due to the demands of caring responsibilities not allowing for sufficient flexibility demanded by employer)
Facilitators	3	1 male (late career), a consultant clinical psychologist. 2 females (early/mid‐career), principal psychologists. All working within community learning disability teams in an NHS Foundation Trust in the North East of England
Academic Researchers	2	1 female (late career), a university professor, with extensive experience of participatory approaches to research and also working with families as a community‐based teacher. 1 female (early career), a senior research assistant, with a clinical background in psychology and learning disability services

### 4.1 Pre‐course semi‐structured interviews (recorded)

These were held prior to the start of the course with consented family carers and course facilitators to ascertain current understandings of their life or work situations and roles, their expectations of the course and what would signify the success of the course for them (Table 3).

**Table 3:** Participation across research dimensions

	Deciding research focus	Designing research methodology and method	Data generation	Data analysis	Taking action	Report writing	Dissemination
Co‐option							
Compliance							
Consultation	X	x				x	
Co‐operation	X	x	X	x		x	x
Co‐learning		x	x	x	x		x
Collective Action			x	x	x		

Key	
Co‐option	Token representatives are chosen but have no real input or power in the research process
Compliance	Outsiders decide the research agenda and direct the process, with tasks assigned to participants
Consultation	Local opinions are asked for, but outside researchers conduct the work and decide on a course of action
Co‐operation	Local people work together with outside researchers to determine priorities, with responsibility remaining with outsiders for directing the process;
Co‐learning	Local people and outsiders share their knowledge to create new understanding and work together to form action plans—facilitation provided
Collective action	Local people set their own agenda and mobilise to carry out research in the absence of outside initiators and facilitators

Note

Adapted from the work of Cornwall A. Unpacking ‘Participation’: Models, Meanings and Practices, *Community Development Journal* **43(**3) 2008; 269‐283 and Cook T, Boote J, Buckley N, Vougioukalou S, Wright M. Accessing Participatory Research Impact and Legacy: Developing the evidence base for participatory approaches in health research*. Educational Action Research* 2017; **25**(4) 473‐488.

### 4.2 Communicative spaces

Communicative spaces were places for “authentic participation”^24^; spaces where people came together for “…mutual recognition, reciprocal perspective taking, a shared willingness to consider one's own conditions through the eyes of the stranger, and to learn from one another”.^25^ The aim was not to strive for consensus based on current knowledge, but to bring together, and wrestle with, “mutually incompatible alternatives”.^26^ This required the cultivation of an ethos of critical thinking, of “openness, receptivity, sensibility and critical reflection upon our assumptions, limitations, blind spots and discourse”.^19^ The communicative spaces were positioned to allow reflexive scrutiny of new understandings by all those taking part. This recursive approach was the key means for generating and analysing data and the springboard for “the construction of knowledge with its enactment in practice”.^27^

Designing safe spaces for reflexive practice was important given the perceived hierarchical differences between professionals and family carers, and family carer predominantly negative perceptions of service provision. The need to be critical within the research process was introduced to family carers during discussions at the start of each course. It was framed as a way of supporting future families (a motivation for participation revealed during the initial interviews). If family carers were not honest about how the course was working, and said things were fine when they were not, it would perpetuate the cycle of family carers coming to ineffectual courses. This encouraged those who might be nervous of being critical, especially of those perceived as having expertise or power over them, to make their voices heard (not all family carers were, however, shy of being critical).

The communicative spaces originally took place directly after session 1, 3 and 5 of each course. This was a pragmatic response to the learning from the pre‐course discussions where family carers had specifically expressed the need to minimize time away from home. The spaces, facilitated by the two academic researchers, were designed to:

- allow different interpretations/understandings to be surfaced and discussed
- learn with and from each other
- develop further discussion topics
- check meanings made from the previous week's data (data analysis) held resonance for group members (face validity and triangulation).
- integrate learning from the meaning making.

Originally standing outside the main body of the course, the communicative spaces had, by the final course, become embedded into the core of the sessions. They were no longer merely a research process but also an extra layer of self‐reflection that re‐enforced and deepened understandings developed through the Mindfulness/ACT programme.

Discourse during the communicative spaces was recorded, transcribed, lodged in NVIVO and thematically analysed by the two academic researchers. First, they undertook this separately and then came together to discuss their understandings of the analysis, taking a specific interest in any differences in meaning making. They fed their understandings back to the group the following week as a starting point for further whole‐group meaning making and discussion (especially about any diverse understandings and what could be learnt from those). In this way, people came back to the initial discussion topics several times, seeing them again through the new lens formed by mutual critical reflexivity.

### 4.3 Post‐course communicative spaces and interview

After each course, all involved (family carers and facilitators) were invited to meet to discuss what had been important for them during the course and how they had used any learning from their experiences during the sessions to make changes in their lives or professional practice. The meetings took place 6‐12 weeks after each course, depending on the availability of family carers. The gap between the end of the course and this meeting allowed time for new ways of acting to be absorbed into daily life. If people could not attend the meeting, individual interviews were held.

### 4.4 Meta approach for collaborative meaning making (data analysis)

Once all courses had been completed, further collaborative group analysis was undertaken. The aim was to critique themes already drawn from each course and to identify any further meanings emerging from a meta‐synthesis of data with people who had taken part in any of three courses. Bringing together people from different courses held the potential to bring different insights to the data.

### 4.5 Recruitment

The key criteria for family carer recruitment were that the person they cared for was over 18 years old and had a learning disability and behaviour that challenged. Family carers were reached through voluntary organizations (particularly family carers' centres), through organizations attended by their family members or through connections with the NHS Trust (family carers were not patients so could not be recruited directly through Trust databases). One family carer who had no connection to organizations, or to other family carers, came to the project after seeing it advertised on social media. Some family carers, having completed the course, used word of mouth to help recruitment to subsequent courses. The facilitators were all recruited due to their role in the course. Had they not wanted to take part in the research, they would still have facilitated but their involvement would not have been used for research purposes.

All family carers completed the course, although attendance was sometimes interrupted by emergency family issues. Two family carers began a course but had to drop out before the end due to such emergencies, but both re‐joined later courses and completed the sessions.

## 5 FINDINGS

Findings from this research emerged throughout the action research process. This was because the communicative spaces, where shared data generation and meaning making led to on‐going learning that directly affected the behaviours/actions of family carers and professionals, happened throughout. Writing about this conflation element of participatory research Wadsworth stated^28^:

…while there is a conceptual difference between the “participation”, “action” and “research” elements, in its most developed state these differences begin to dissolve in practice…there is not participation followed by research and then hopefully action. Instead there are countless tiny cycles of participatory reflection on action, learning about action and then new informed action which is in turn the subject of further reflection. Change does not happen at “the end”—it happens throughout.

Presented below are firstly findings in relation to behavioural changes instigated through the processes of the course and secondly the impact of the PHR approach on course content and processes.

### 5.1 Behavioural change: family carers

For most family carers, the effects of this short course were rapid, reportable and observable. 14/18 family carers reported making changes to their own behaviours that improved their mental (and in some cases physical) health during or after the course, and associated improvements in family life. Mindfulness practices such as mindful walking, or mindful eating, that did not need a special time or place, meant that family carers could, and did, practice them. Enabling people to maintain regular mindfulness practice (even if short) was a major factor for both short‐ and longer‐term (3 months plus) change. For many, enjoyment and their own nourishment had been missing from their lives and they began to prioritize time to do things they took pleasure in. For most, it started small, making commitments to invest in 15 minutes for themselves to read a book, do a mindfulness exercise or simply be alone. Some joined choirs and made time to go to cafes or time to go out with their husbands/wives/partners/friends and even their family members whose behaviour had gradually led them to constrict their own lives.

**Figure 2:**
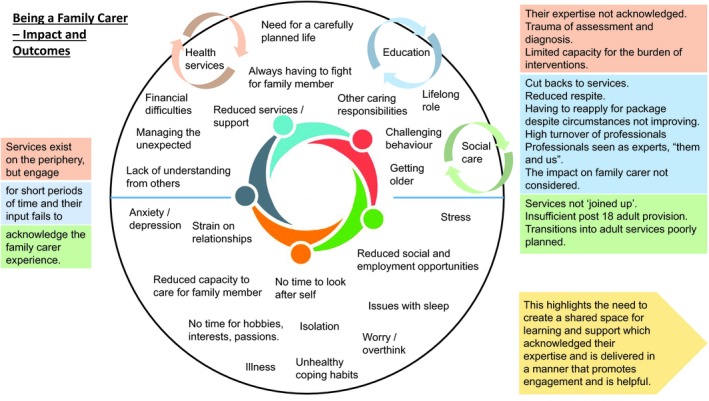
Complex lives of family carers

The initial interviews revealed the complex lives of family carers (Figure 2).

Stressful lives had led people to construct rigid notions of what they “ought” to do. Some found it difficult to let go of unnecessary elements and become embroiled in self‐imperated knots.^29^ Using metaphor of the “Prison of Oughts” introduced by a course facilitator, this family carer explained how they had used it to reduce their anxiety.

I sometimes use the Ought Prison…I use it to write a list of all the things I ought to be doing…writing lists and really thinking about it …[means I can say] No – that's not my problem. I can delegate that one, or delete it, and you're left with two or three at the end. C^1^2:FC^2^12.

He/she also used the “Thought Train” to “shove all those [stressful] thoughts on the train and move them on to the next station”. This took “some of it [stressful thoughts] away and only the important ones seem to come back”. C2:FC12.

A metaphor of “Mindblank” helped this family carer to see and address the way in which he/she was medicating their stress with alcohol.

I do take tablets for high blood pressure, and I shouldn't be drinking as much wine. So, it's making me think about myself in a [new way]. I didn't have anyone to sort of…say to me… I got that thought here, last week…I did it for a week [didn't drink]…I thought, yeah, this is life changing, really, what I'm thinking. And I went home and didn't have any alcohol all week…I could get through five bottles of wine a week. C2:FC12.

Some family carers reported reducing long‐term medication they had been taking for stress after being part of the course, others reported the reduction in medication for the person they cared for because, as they were now calmer, so was their family member.

Changes in long‐term, unhelpful, response behaviours that perpetuated the challenging behaviours of family members, creating more stress, were revealed and recounted. This family carer recognized that matching threat with threat in challenging circumstances (“I used to threaten him and say “I'll hit you back if you hit me” PCCS1^3^ :FC5”) had ratcheted‐up the situation. Through talking about this within the group, he/she realized that he/she did not do that now. Being calmer in the situation also had a ripple effect on their family member. “I don't threaten him now and I think that's perhaps one of the reasons why he's calmed down much more. Because there's not that conflict”. PCCS1:FC5.

Instigating “thinking before acting” as their response behaviour enabled this family carer to avoid a previously experienced challenging situation when the shop their son wanted to go to was unexpectedly closed.

I just got him back to the car… I said to [spouse] “Just drive” He [son] was getting really agitated, but I thought, no, he's in the car, he can't do anything. So, we went round the corner and I said to [spouse], “Get in that car wash.” So, we just sat in the car wash … he [son] was really calm … then we went back to the shop and everything was alright. There was no raised voices…Usually, I go, “Oh, God, no…what are we going to do?” [panicky voice] … it was great. Normally I would be pounding. C1:FC3

Family carers devised their own ways of acting and re‐acting, constructing new response behaviours to fit their own circumstances in their own way and in their own time. They made changes not because external evidence presented a case to them for change, nor because of suggestions/directions made by facilitators. This was a self‐actualized response, drawn out of collaborative discussions, and was key to the effectiveness of the course.

What has been different here is that it is not all give [from facilitators] and take [by family carers] we've not been told. We have done it in this group – everyone has chipped in and we find out from each other. C2:FC10.

### 5.2 Impact of the participatory approach

The process of shared recursive reflections and actions enabled people to see differently and find spaces to affect their own actions. This was powerful not only for those whose voices were seldom heard and even more seldom afforded agency in change processes, but also for those who generally found themselves in the position of offering their professional knoweldges as expertise.

#### 5.2.1 Impact on course content

Consultations with family carers prior to designing the project showed how the concept of Mindfulness/ACT was seen as alien, and hence not relevant, for most people. In addition, because the course focused on self‐nourishment, and not the needs of the person they cared for, it tended to be viewed as less important. This led to the initial intention to include an element in the course that would focus on supporting family members, positive behaviour support (PBS). PBS seeks to understand the reasons for behaviours exhibited by people with learning disabilities and focuses on the teaching of new skills to replace such behaviours.^30^ As they became more deeply involved in the FaBPos project, however, family carers and facilitators recognized how family carers were re‐prioritizing their need for self‐nourishment, even those who had initially come seeking help for building their resilience. Seeking self‐nourishment came to be recognized not only as a way of keeping healthy enough to be able to continue to provide for family members, but also intrinsically important for themselves. The outcome was that as the course evolved, this became a topic for discussion and led family carers to consider PBS as irrelevant for this particular course.

Pre‐course interviews had revealed the difficulties family carers had in suggesting what might support them in their caring role beyond having the opportunity to share their knowledge and experience with other family carers and in turn learn from others. They could not envisage other elements for a course that would support them. Facilitators on the other hand had a relatively clear idea of how a course might work. Had the knowledge of both parties merely been collected and synthesized the course would have been founded on prioritization of previous experience and knowledge. Given the dominance of professional knowledge, the danger was that this strengthened the power of that knowledge in shaping practice. To truly learn, knowledge had to be disturbed rather than maintained. Merely collecting undisturbed experiences and knowledge would not create new ways for understanding and acting. The “…sustained *collective deliberation* coupled with sustained *collective investigation”* of the research process enabled people to further “explore possibilities in action”*.* 32 Honest and open critique disrupted ways of thinking that had become embedded as given wisdom, whether that was wisdom from lived, or professional experience. This built on the principles of collective thinking that underpin Mindfulness/ACT processes, adding the disruptive element of critical thinking that became pivotal for re‐shaping the course using the collective wisdom of those engaged.

One of the big breakthroughs…what's been great, is doing it together [as] what we've ended up with is different from what we started off with [We've] created something that none of us would have thought of if we had not gone through it. PCI^4^:FacA.

#### 5.2.2 Impact on professional learning

During course discussions, criticism of professionals that, in their clinical roles, psychologists were unlikely to hear was voiced.

Normally, in my role as a clinical psychologist, people don't say that to me. Because, I guess, they might feel I might be offended or… it's just not something people would say. But, in that forum, people were really candid about their experiences PCI:Fac^e^B

Due to such honesty, those whose knowledge traditionally dominated were challenged by hearing other perceptions of the impact of their practice. To learn something new about a practice in which you already have considerable expertise is not easy for any party. Reflecting on their practice in this way led facilitators to “think about what sort of professional you do want to be” PCI:FacB. That would mean having to let go of some of your own ideas/beliefs about those practices.^33^ A challenge for professionals in doing this was the strength of their own professional expectations.

‘We’re brought up on training and not…facilitating'. Not being in control in the traditional way creates anxiety … my default position, when I feel like that, is to over prepare…to have an agenda…now I'm really conscious of… how I manage my own anxieties, so I can be less in control, and let it be led by families, rather than myself PCI:FacB

Their willingness to consider their own ways of acting in the light of critique led facilitators to personal epiphanies.

It's so easy for us as professionals to think these are the latest psychological benefits. We should make them available. Which is a decent start. But how you go about making them available is you do unto them. I think one of the things that we've learnt in this course is you don't do unto them. That's so crucial. So, dismantle the doing unto PCI:FacA.

As with family carers, facilitators were not told, or asked, to change their behaviours. This emerged from the reflexive processes for critical thinking embedded in the research process and became something they wanted/needed to initiate for themselves.

Personal learning and change addressed some of the issues that, in the past, had led family carers to perceive professionals as “all give and no take” C2:FC10. Facilitators needed a high level of knowledge of Mindfulness/ACT, but facilitating the course as a relationally based shared learning space was fundamental to its success. Central to family carer involvement was that services listened to them. In session 1, course 1, family carers challenged facilitators about practices that unwittingly shaped and led the agenda for the course and alienated family carers. The starting point had to be the act of listening to family carer stories. Only then, serendipitously, could facilitators introduce Mindfulness/ACT practices to aid deeper thinking about the issues raised by those stories. Facilitators had to take their cue from family carers. What emerged was a process termed by one family carer as “invisible facilitation”. Affecting “…understanding of these practices and the situations in which these practices are carried out”^34^ is at the root of PHR.

#### 5.2.3 Impact on research methods

The initial design for the project had been determined by the consultations with family carers and using the expertise of the PDT. Key changes to the research approach and course design continued, however, throughout the project. For example, in a post‐course feedback session, to try and elicit which materials in the course were working for family carers, the academic researchers deviated from the communicative space approach. They introduced an exercise for family carers to sort materials used during the course into the categories “most used”, “used” and “least used”. The subsequent silence was broken by one family carer saying this could not be done. Knowing and discussing the concepts behind all the materials had enabled this person to make substantial changes to their life, but he/she did not use any of the materials. To categorize them as “not useful” was not helpful and he/she did not want to do it as he/she believed that then other family carers would miss out by their omission in future courses. Reflecting together on this made it clear that the academic researchers had presented a “task” that did not fit with the participatory ethos of the project. The weight of historical teaching about the need for more definitive research approaches had led them to look for a concrete representation of evidence. The established participatory approach for critique within the project thus extended to the research approach. Reasons why this approach had not worked were surfaced, and the research agenda was reset to the discursive approach central to the course and to PHR. This led to more meaningful understandings about the way in which materials used during the course had impact for family carers that would have been lost if the more traditional “test” method had been accepted without critique.

## 6 DISCUSSION

In the FaBPos project, the involvement of facilitators as researchers into their own practice was fundamental for effective change. Institutional and professional capacity for change has been recognized as an obstacle to achieving changes needed to enhance health and well‐being. Institutional relations are often rather closed to the engagement of other actors.^35^ This enhances existing cleavages that contribute to distrust in both processes and outcomes instigated by institutions. Communicative spaces are not only spaces where data are generated and analysed but act as change mechanisms. Co‐creating self‐knowledge through co‐labouring is a powerful tool. It was, therefore, essential in the FaBPos project to have communicative spaces that bridged diverging interests and perceptions and mobilized different knowledges and expertise for change. The PHR approach served to break down the more traditional notion of research “where professional knowledge is separated from, and valued above, situational, visual and experiential knowledge”.^19^

In PHR, the multiple perspectives and recursive opportunities for data generation and analysis, learning and action, confer strength, meaning and (to borrow a word from a more positivist paradigm) validity.^33^ Different knowledges are recognized and valued as legitimate. If one person remains aloof as a so‐called “objective” observer, this would have risked “…the worst kind of subjectivism – the objective observer is likely to fill in the process of interpretation with his own surmises in place of catching the process as it occurs in the experience of the acting unit which uses it”.^36^ This can be a challenge to those who see the role of the social scientist as identifying and establishing facts, presenting and testing hypotheses, and use research processes that follow established methods where one's personal views are put to one side.

In the FaBPos project, family carers and facilitators shaped the design and carried out the systematic enquiry and meaning making through disturbing their own and each other's assumptions and learning new ways together. Sharing responsibility for the creation of the new course was described by this family carer as crucial, “you don't know how important this is” C2: FC9. During the project, there were, however, times when everyone could not be part of the whole process. Family carers from the initial PDT were not able to engage in the main project, family carers from each of the first two courses did not want to become research active in subsequent courses and most were reluctant to be part of the dissemination strategy. Their full, complex and hectic lives meant they could only be involved in the course/research where it had direct meaning for them. The widely held assumption that to be termed PHR (or participatory action research) necessitates full participation in all elements tested our assertion that the research process underpinning the FaBPos project was indeed PHR. The following table shows the dimensions for participation in the FaBPos research.

PHR can take many forms, but it is necessary to be clear, and make clear, how the values that underpin PHR are played out in practice. The marker for PHR in the FaBPos project was being faithful to the values of shared knowledge creation and agency. Driven by a form of engagement that created spaces for valuing experiential and professional knowledge, the starting point for this research (systematic, collaborative critique) was the catalyst for joint decision and effective change. Communicative spaces challenge more traditional health research paradigms where the research process, and what can be documented and valued as outcomes, is decided upon by outside researchers. If how well we respond to the challenge of bringing knowledges together determines the quality of research results for both societal and scientific praxis,^37^, ^38^ then the FaBPos project rose to that challenge. Those involved systematically generated, and critiqued, self and collective knoweldges. This created spaces for new knowledge, learning and actions. Concerns that FaBPos was not PHR, which ladders or models for participation demonstrated gaps in full participatory practices, reiterate the importance of having principles and values but not fixed ideas that drive a PHR design.

## 7 REFLECTIONS

The process of undertaking this research has taught much about what makes an effective course for family carers. It has also taught much about how people can, and need to be, involved in that process. It has highlighted that what determines quality is not a set of practices, although practices are obviously part of the mix, but the set of overt principles and values for practice that ultimately determine the quality of practice and the quality of research processes. This has implications for the education of professionals working and researching with families per se and for further research into the effects of the current professional‐expert‐driven model on the effectiveness of services for families with long‐term caring roles. Many social institutions such as health and social care have created services to support people by focusing on perceived needs and deficiencies rather than identifying and fostering the skills and resources of those with lived experience.^19^ To make a difference to current systems, it is important to go beyond the prioritization of individual knowledges brought to the table and to recognize collective disruption of knowledges as the basis for new learning and effective change. This includes the disruption of frameworks for research quality that priorities pre‐determined tools and measures for collecting undisturbed data.

Disruption of powerful systems necessitates the production of new narratives that involve those who are already in places of power working alongside those whose voices have not been heard, have been under‐represented or have been heard without agency. This highlights the importance of facing the challenge of integrating the co‐construction of knowledge with its on‐going enactment in practice. This is not a passive act but a radical engagement where the “need to disrupt” is a central element for addressing traditional power imbalances and building pathways for democratic change. This is not an easy or static process “…it's like building an aeroplane while we're flying it”. C2:FacA.

## NOTES

1 C denotes course.

2 FC denotes family carer.

3 PCCS denotes post‐course communicative space.

4 PCI denotes post‐course interviews.

5 Fac denotes facilitator.
